# Borophene and Pristine Graphene 2D Sheets as Potential Surfaces for the Adsorption of Electron-Rich and Electron-Deficient π-Systems: A Comparative DFT Study

**DOI:** 10.3390/nano12061028

**Published:** 2022-03-21

**Authors:** Mahmoud A. A. Ibrahim, Amna H. M. Mahmoud, Kamal A. Soliman, Gamal A. H. Mekhemer, Muhammad Naeem Ahmed, Ahmed M. Shawky, Mohammed A. S. Abourehab, Eslam B. Elkaeed, Mahmoud E. S. Soliman, Nayra A. M. Moussa

**Affiliations:** 1 Computational Chemistry Laboratory, Chemistry Department, Faculty of Science, Minia University, Minia 61519, Egypt; a.mahmoud@compchem.net (A.H.M.M.); gmekhemer@mu.edu.eg (G.A.H.M.); n.moussa@compchem.net (N.A.M.M.); 2 Department of Chemistry, Faculty of Science, Benha University, Benha 13518, Egypt; kamal.soliman@fsc.bu.edu.eg; 3 Department of Chemistry, The University of Azad Jammu and Kashmir, Muzaffarabad 13100, Pakistan; drnaeem@ajku.edu.pk; 4 Science and Technology Unit (STU), Umm Al-Qura University, Makkah 21955, Saudi Arabia; amesmail@uqu.edu.sa; 5 Department of Pharmaceutics, Faculty of Pharmacy, Umm Al-Qura University, Makkah 21955, Saudi Arabia; maabourehab@uqu.edu.sa; 6 Department of Pharmaceutical Sciences, College of Pharmacy, AlMaarefa University, Riyadh 13713, Saudi Arabia; ikaeed@mcst.edu.sa; 7 Molecular Modelling and Drug Design Research Group, School of Health Sciences, University of KwaZulu-Natal, Westville, Durban 4000, South Africa

**Keywords:** 2D nanomaterials, borophene, pristine graphene, aromatic π-systems, adsorption energy, electronic properties

## Abstract

The versatility of striped borophene (sB), *β*_12_ borophene (*β*_12_), and pristine graphene (GN) to adsorb π-systems was comparatively assessed using benzene (BNZ) and hexafluorobenzene (HFB) as electron-rich and electron-deficient aromatic π-systems, respectively. Using the density functional theory (DFT) method, the adsorption process of the π-systems on the investigated 2D sheets in the parallel configuration was observed to have proceeded more favorably than those in the vertical configuration. According to the observations of the Bader charge transfer analysis, the π-system∙∙∙sB complexes were generally recorded with the largest contributions of charge transfer, followed by the π-system∙∙∙*β*_12_ and ∙∙∙GN complexes. The band structures of the pure sheets signaled the metallic and semiconductor characters of the sB/*β*_12_ and GN surfaces, respectively. In the parallel configuration, the adsorption of both BNZ and HFB showed more valence and conduction bands compared to the adsorption in the vertical configuration, revealing the prominent preferentiality of the anterior configuration. The density-of-states (DOSs) results also affirmed that the adsorption process of the BNZ and HFB on the surface of the investigated 2D sheets increased their electrical properties. In all instances, the sB and *β*_12_ surfaces demonstrated higher adsorptivity towards the BNZ and HFB than the GN analog. The findings of this work could make a significant contribution to the deep understanding of the adsorption behavior of aromatic π-systems toward 2D nanomaterials, leading, in turn, to their development of a wide range of applications.

## 1. Introduction

Graphene, a single-atom-thick layer of sp2-bonded carbon atoms tightly packed into a two-dimensional (2D) honeycomb lattice, has been become the linchpin of a variety of fields in science and technology, especially electronics [1,2], condensed-matter physics [3], biomedical research [4,5,6], and energy storage [7,8,9]. Immense efforts were therefore undertaken to fully uncover the characteristics of graphene and its derivatives. For graphene, or, to be more precise, pristine graphene (GN), various superior physical and chemical properties, including large surface area, mechanical strength, fascinating thermal and electrical conductivity, have hitherto been deeply understood and well established through significant studies [10,11,12]. Consequently, GN has been widely used in various industrial applications, such as sensors, lithium-ion batteries, and catalysis [13,14,15,16]. The utilization of GN in removing toxic volatile organic compounds (VOCs) such as benzene, pyridine, and thiophene from the air was also reported [17,18].

Soon after the recognition of GN, sustained interest developed in the detailed investigation of the features of elemental 2D materials, including germanene [19,20], phosphorene [21,22], transition metal dichalcogenides [23,24], and silicone [25,26], using advanced experimental and computational techniques. Crucially, borophene—a two-dimensional boron sheet—has recently drawn an upsurge in attention from the scholar community due to its promising properties, such as its preferential surface reactivity, unique electronic properties, and high electrical conductivity [18,27,28,29,30]. Moreover, some borophene structures have been predicted to host Dirac fermions [31]. The applications of borophene have been extended to lithium-ion batteries, hydrogen detection, and hydrogen storage [32,33,34]. Experimentally, borophene was successfully grown on an Ag(111) surface under ultra-high-vacuum conditions [27,29].

Using high-resolution scanning tunneling microscopy (STM), different borophene phases were noticed, such as striped (sB), *β*_12_, and χ_3_ phases [27]. As previously demonstrated, the *β*_12_ and *χ*_3_ phases of borophene showed more obvious stability than the sB phase, which was interpreted as a consequence of their planar structures, which had 1/6 and 1/5 vacancies, respectively [35,36,37,38,39,40,41]. Upon examining its mechanical and thermodynamical features, *β*_12_ borophene was found to be the most stable phase of borophene [38,42]. Significantly, the sB phase of borophene demonstrated anisotropic metallic behavior through its unique buckled structure [27,28]. Owing to its anisotropic character, the sB was found to be stiffer than GN along one axis (i.e., *a* direction) [27,42,43]. Nonetheless, along *a* direction, the sB demonstrated phonon instability [28], while elastic instability was observed along the *b* direction [43].

**Figure 1:**
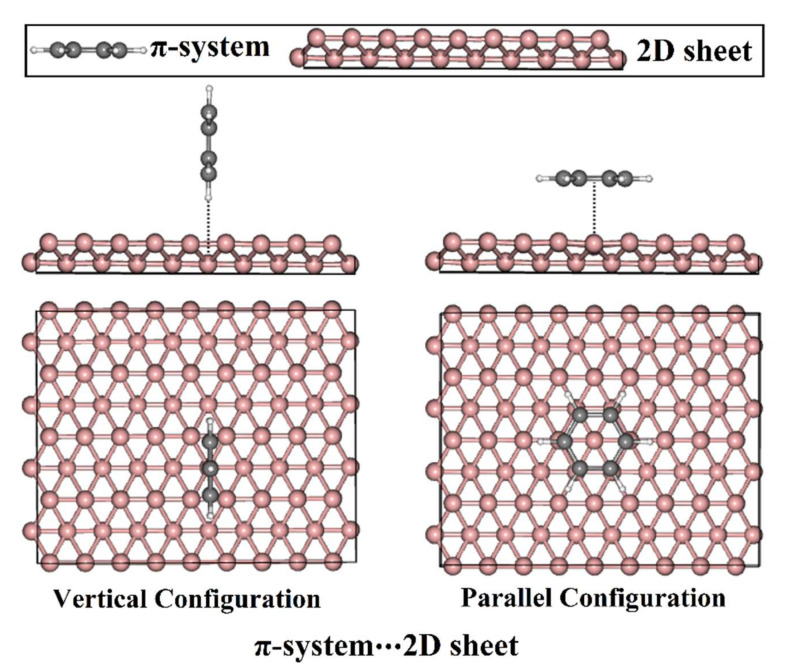
Side and top views of the vertical and parallel configurations of the π-system∙∙∙2D sheet complexes where π-system = BNZ/HFB and the 2D sheet = sB/*β*_12_/GN.

As an essential issue, the aromatic π-systems were recorded as volatile organic compounds (VOCs) that were emitted from vehicle exhaust, petrochemical technologies, and thinners [44,45]. These compounds were found to have significant toxicity and carcinogenic effects on public health [46,47]. Numerous studies were accordingly designed to limit the existence of π-systems through the adsorption process on the surfaces of efficient 2D nanomaterials, such as stanene, CrI_3_, GN, χ_3_ borophene, *β*_12_ borophene, and germanene [48,49,50,51,52,53,54,55,56]. A careful literature search revealed that the adsorption of electron-rich and electron-deficient aromatic π-systems in different borophene phases and GN sheets has not been sufficiently studied yet. Accordingly, the present work is devoted to thoroughly investigating the adsorption of the electron-rich and electron-deficient aromatic π-systems on anchoring 2D sheets by means of the density functional theory (DFT) method. To pursue the aim of the current work, benzene (BNZ) and hexafluorobenzene (HFB) were utilized as electron-rich and electron-deficient aromatic π-systems to be adsorbed at all possible sites of adsorption on the surface of the sB, *β*_12_, and GN sheets in vertical and parallel configurations (Figure 1). For the studied π-system∙∙∙2D sheet complexes, the analyses of geometric structures and the adsorption energies were performed. Furthermore, the electronic properties, including charge transfer, band structures, and density of states (DOSs), were precisely assessed for the 2D sheets before and after the adsorption process. The obtained findings will serve as the foundation for future research pertinent to the application of borophene and graphene as adsorbent materials.

## 2. Computational Methods

All calculations were performed with Quantum ESPRESSO 6.4.1 package [57,58] using density functional theory (DFT) [59,60]. Generalized gradient approximation (GGA) was employed using the Perdew–Burke–Ernzerhof (PBE) exchange-correlation function [61]. The ultrasoft pseudopotential was adopted for treating electron–ion interactions [62]. The DFT-D2 method of the Grimme scheme [63] was applied to account for the van der Waals interactions in all computations. The cutoffs of energy and charge density were set to be 50 Ry and 500 Ry, respectively. All structures were fully optimized with respect to the energy convergence threshold of 10^−5^ eV and force convergence threshold of 10^−4^ eV/Å. Monkhorst–Pack grids of 6 × 6 × 1 and 12 × 12 × 1 *k*-points were used to sample the first Brillouin zone for geometric optimization and electronic structure calculations, respectively, combined with the Marzari–Vanderbilt smearing technique [64]. A vacuum of 20 Å was settled along the vertical direction of the borophene and the graphene surfaces for all calculations to avoid spurious interactions between the periodic cells in the *z*-direction. The corresponding space groups of the studied sB and *β*_12_ sheets were Pmmn and P2mm, respectively.

The potential of sB, *β*_12_, and GN to adsorb BNZ/HFB as electron-rich/electron-deficient aromatic π-systems was comparatively assessed. For adsorption energy calculations, supercells of 9 × 4 × 1, 3 × 4 × 1, and 6 × 5 × 1 were constructed for sB, *β*_12_, and GN, respectively. The number of atoms in the supercells of sB, *β*_12_, and GN was 72, 60, and 60, respectively. On the designed 2D sheets, BNZ and HFB were subjected to adsorption process as electron-rich and electron-deficient aromatic π-systems, respectively, within vertical and parallel configurations (Figure 1). Adsorption energy (*E*_ad_) was computed according to the following equation:

(1) $$E_{\mathrm{ad}\;}=E_{\pi -\mathrm{system}\cdot \cdot \cdot 2D\;\mathrm{sheet}}-\left(E_{\pi -\mathrm{system}}+E_{2D\;\mathrm{sheet}}\right)$$

where $E_{\pi -\mathrm{system}\cdot \cdot \cdot 2D\;\mathrm{sheet}}$, $E_{\pi -\mathrm{system}}$, and $E_{2D\;\mathrm{sheet}}$ were the energies of complex, adsorbed π-system, and anchoring 2D sheet, respectively. Using Bader charge analysis [65,66], the charge transfer (*Q*_t_) to or from the 2D sheets was assessed as follows:

(2) $$Q_{t\;}=Q_{\mathrm{combined}\;2D\;\mathrm{sheet}\;}-Q_{\mathrm{isolated}\;2D\;\mathrm{sheet}\;}$$

where the *Q*_combined 2D sheet_ and *Q*_isolated 2D sheet_ were the charges of the 2D sheet after and before the adsorption process, respectively. Based on Bader charge analysis, the charge density difference (∆*ρ*) was evaluated according to the following equation:

(3) $$\Delta \rho =\rho_{\pi -\mathrm{system}\cdot \cdot \cdot 2D\;\mathrm{sheet}}-\left(\rho_{\pi -\mathrm{system}}+\rho_{2D\;\mathrm{sheet}}\right)$$

where $\rho_{\pi -\mathrm{system}\cdot \cdot \cdot 2D\;\mathrm{sheet}}$, $\rho_{\pi -\mathrm{system}}$, and $\rho_{2D\;\mathrm{sheet}}$ were the charge of the complex, adsorbed π-system, and anchoring 2D sheet, respectively. The charge density difference maps were visualized using the VESTA [67] package. Furthermore, the electronic band structure, total density of states (TDOSs), and projected density-of-states (PDOSs) calculations were thoroughly studied to understand the electronic properties of the studied 2D sheets.

## 3. Results and Discussion

### 3.1. Geometric Structures

**Figure 2:**
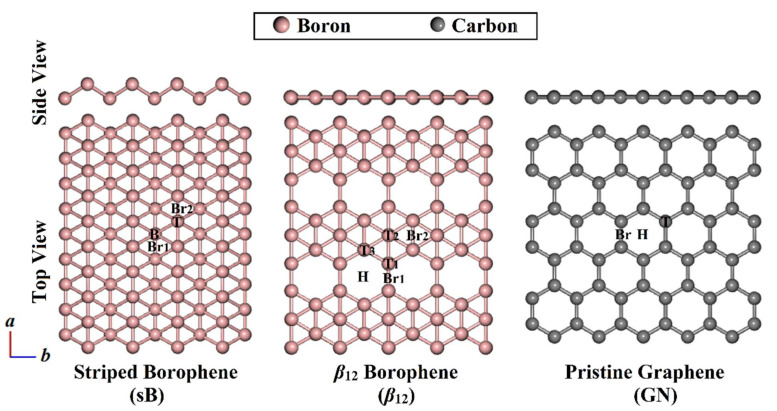
Side and top views of the sB-, *β*_12_-, and GN-optimized 2D sheets. Top (T), bottom (B), and two bridge (Br1 and Br2) sites are labeled on the surface of the sB sheet. On the *β*_12_ surface, three top (T1, T2, and T3), one hollow (H), and two bridge sites (Br1 and Br2) are depicted. Three adsorption sites, namely, top (T), hollow (H), and bridge (Br) sites are illustrated on the GN surface.

Prior to the adsorption calculations of the aromatic π-systems on the 2D sheets, the structures of the striped sB, *β*_12_, and GN were constructed and geometrically minimized to obtain the equilibrium geometries. The optimized structures of the 2D sheets are illustrated in Figure 2.

According to the optimized structures demonstrated in Figure 2, the equilibrium lattice constants (*a* × *b*) of sB and *β*_12_ were 1.64 Å × 2.93 Å and 5.06 Å × 2.93 Å, respectively. For GN, the calculated lattice constant values of the unit cell were 2.47 Å × 2.47 Å. The abovementioned values of the equilibrium lattice constants of sB/*β*_12_ and GN were consistent with the values reported by the literature, which were 1.61 Å × 2.86 Å/5.0 Å × 3.0 Å [27,28,29] and 2.46 Å × 2.46 Å [17,51], respectively.

Because of the buckled structure of the sB with corrugations along the *b* direction, two types of boron atoms, namely, T and B atoms, were observed, as depicted in Figure 2. The calculated T–T and B–B distances were 1.61 Å. The T–B distance was 1.88 Å, and the buckling height was 0.92 Å. All the measured distances were consistent with the literature [28,32,43]. Accordingly, the sB sheet was found to have four different adsorption sites, termed the top site (T@sB), bottom site (B@sB), and bridge sites (Br1@sB and Br2@sB) (Figure 2).

Based on the geometrical position of the boron atoms, three different boron atoms –namely, T1, T2, and T3—were observed in the *β*_12_ sheet (Figure 2). The T1–T1, T1–T2, and T1–T3 bond lengths were 1.65, 1.71, and 1.68 Å, respectively. These different bonds led in turn to the existence of six possible adsorption sites on the *β*_12_ sheet; three top (T1@*β*_12_, T2@*β*_12_, and T3@*β*_12_), one hollow (H@*β*_12_), and two bridge sites (Br1@*β*_12_ and Br2@*β*_12_). When observing the GN sheet, a symmetric carbon–carbon bond was detected with a distance of 1.42 Å, resulting in three adsorption sites—namely, top (T@GN), hollow (H@GN), and bridge (Br@GN).

### 3.2. Adsorption Energy Calculations

**Figure 3:**
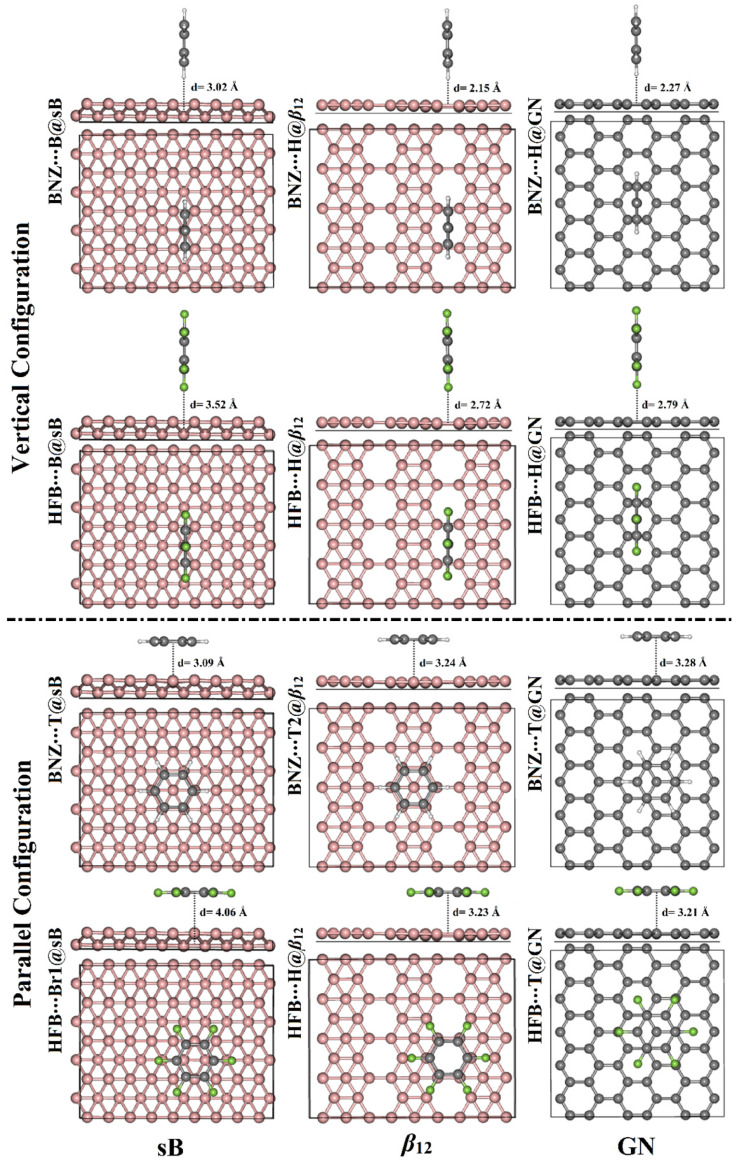
Side and top views of the relaxed structures of the vertical and parallel configurations of the π-system∙∙∙2D sheet complexes where π-system = BNZ/HFB and the 2D sheet = sB/*β*_12_/GN at the most energetically favorable adsorption sites. The π-system–2D sheet equilibrium distances (d) are given in Å.

To obtain a better understanding of the adsorption process within the π-system∙∙∙2D sheet complexes, BNZ and HFB were placed at versatile sites on the surfaces of sB, *β*_12_, and GN in both vertical and parallel configurations (Figure 1). Upon the relaxed structures, the adsorption energies were computed according to Equation (1) (see Computational Methods section), and the equilibrium distances were assessed (Table 1). The obtained relaxed structures for all the studied π-system∙∙∙2D sheet complexes are depicted in Figure S1. Figure 3 illustrates the relaxed π-systems at the most favorable energetic sites on the studied 2D sheets.

At first glance, the considered aromatic electron-rich and electron-deficient π-systems demonstrated an obvious tendency to be adsorbed on the relaxed 2D sheets (Figure S1), producing considerable negative adsorption energies (Table 1). The π-system∙∙∙sB, ∙∙∙*β*_12_, and ∙∙∙GN intermolecular distances were found to have values in the range of 2.54–4.06 Å, 2.15–3.30 Å, and 2.27–3.40 Å, respectively.

With regard to the adsorption process of the π-system on the surface of the sB sheet in a vertical configuration, the most prominent adsorption energy was detected in the case of the π-system∙∙∙B@sB complexes, followed by the π-system∙∙∙Br1@sB, π-system∙∙∙Br2@sB, and, finally, π-system∙∙∙T@sB complexes. For instance, the adsorption energies of the BNZ on the surface of the sB sheet at the B@sB, Br1@sB, Br2@sB, and T@sB sites were −5.66, −5.64, −4.40, and −4.35 kcal/mol, respectively. For parallel configuration, the T@sB and Br1@sB sites were found to be the most preferential adsorption sites for the BNZ and HFB, with adsorption energies of −14.93 and −15.76 kcal/mol, respectively.

According to the data registered in Table 1, the adsorption of the BNZ and HFB on the *β*_12_ surface was occurred significantly at the H@*β*_12_ site in the vertical configuration with adsorption energy values of −6.40 and −4.54 kcal/mol, respectively. Turning to the parallel configuration of the π-system∙∙∙*β*_12_ complexes, the T2@*β*_12_ and H@*β*_12_ sites were found to have significantly negative adsorption energy values in the interactions with BNZ and HFB, respectively.

Similar to the π-system∙∙∙*β*_12_ interactions, the H@GN site was documented as the most promising site for the adsorption of both BNZ and HFB on the GN surface in the vertical configuration. In comparison, the T@GN site was found to have the largest negative adsorption energies of the π-system∙∙∙GN complexes in the parallel configuration, followed by the Br@GN and, finally, H@GN sites. For example, the adsorption energies of the BNZ–GN complexes in the parallel configuration were −9.97, −9.89, and −8.78 kcal/mol for the BNZ–T@GN, BNZ∙∙∙Br@GN, and BNZ∙∙∙H@GN complexes, respectively. The obtained results were found to be compatible with those of an earlier study, demonstrating that the T@GN site was the most favorable site for the adsorption process of BNZ with an adsorption energy of −5.53 kcal/mol [50]. The slight difference between the adsorption energy values in the present study and the preceding study might be ascribed to the different chosen cutoff energies in the calculations.

Notably, the adsorption process of the aromatic π-systems∙∙∙sB/*β*_12_/GN complexes within the parallel configuration was observed to have greater favorability than those in the vertical configuration. For instance, the adsorption energies of the BNZ∙∙∙B@sB complex were −5.66 and −11.46 kcal/mol in vertical and parallel configurations, respectively.

### 3.3. Charge Transfer Calculations

Bader charge analysis has been documented as a powerful tool to precisely obtain an in-depth theoretical insight into the charge transfer during the adsorption process [65,66]. Utilizing Bader charge analysis, the charge transfer differences (*Q*_t_) of the relaxed π-system∙∙∙2D sheet complexes at all possible adsorption sites were calculated as illustrated in Equation (2) in the Computational Methods section, and they are compiled in Table 1. In the context of Bader charge analysis, the negative values of the charge transfer differences (*Q*_t_) outline the transfer of charge from the π-systems to the 2D sheets, and the positive values mean the opposite.

As can be seen from the data in Table 1, the adsorption of HFB on the sB surface had higher negative values of *Q*_t_, compared to BNZ, in the vertical configuration. For example, the *Q*_t_ values were −0.0275 and −0.0154 e within the adsorption process of the HFB and BNZ, respectively, at the T@sB site in the vertical configuration. Although the adsorption of BNZ in the vertical configuration at the B@sB site exhibited the largest negative adsorption energy value of −5.66 kcal/mol, it had a small *Q*_t_ value of −0.0136 e. This observation c confirmed that the charge transfer contributed little to the adsorption process within the BNZ∙∙∙sB complexes in the vertical configuration. On the other hand, the charge transfer in the HFB∙∙∙B@sB complex in the vertical configuration was shown to play the main dominant role in the interaction by the largest *Q*_t_ value (i.e., −0.0318 e). Furthermore, a direct correlation between the *Q*_t_ and the adsorption energy values was observed for the π-system∙∙∙sB complexes in the parallel configuration. For example, the BNZ∙∙∙T@sB and HFB–Br1@sB complexes, the most stable complexes in the parallel configuration with adsorption energy values of −14.93 and −15.76 kcal/mol, showed *Q*_t_ values of 0.0735 and −0.0813 *e*, respectively.

In general, the charge transfer’s contribution to the adsorption process of BNZ and HFB on the *β*_12_ surface, in both vertical and parallel configurations, was signaled by smaller values of *Q*_t_ compared to the π-system∙∙∙sB complexes. For instance, the *Q*_t_ values were −0.0318 and −0.0261 e in the case of the HFB∙∙∙Br1@sB and ∙∙∙Br1@*β*_12_ complexes, respectively, in the vertical configuration. Moreover, it was noted that the adsorption of HFB on the *β*_12_ surface resulted from the transfer of a larger amount of the *Q*_t_ compared to the BNZ analog. For example, the BNZ∙∙∙ and HFB∙∙∙H@*β*_12_ complexes in the vertical configuration had *Q*_t_ values of −0.0199 and −0.0274 e, respectively.

Notably, the *Q*_t_ showed small values, ranging from −0.0058 e for the BNZ∙∙∙GN complexes to −0.0124 e for HFB∙∙∙GN complexes, indicating the small contributions of charge transfer to the adsorption process on the GN surface in the vertical configuration.

Interestingly, in the case of parallel configuration, the adsorption of BNZ on all the studied 2D sheets showed positive *Q*_t_ values. For example, the *Q*_t_ values were 0.0735, 0.0436, and 0.0065 *e* for the BNZ∙∙∙T@sB, ∙∙∙T2@*β*_12_, and ∙∙∙T@GN, respectively. The latter observation emphasized that the charge was transferred from the 2D sheets to the BNZ. Similar findings of small positive *Q*_t_ values in the case of the adsorption of BNZ on the GN surface in the parallel configuration were observed in prior theoretical studies [49,50].

**Figure 4:**
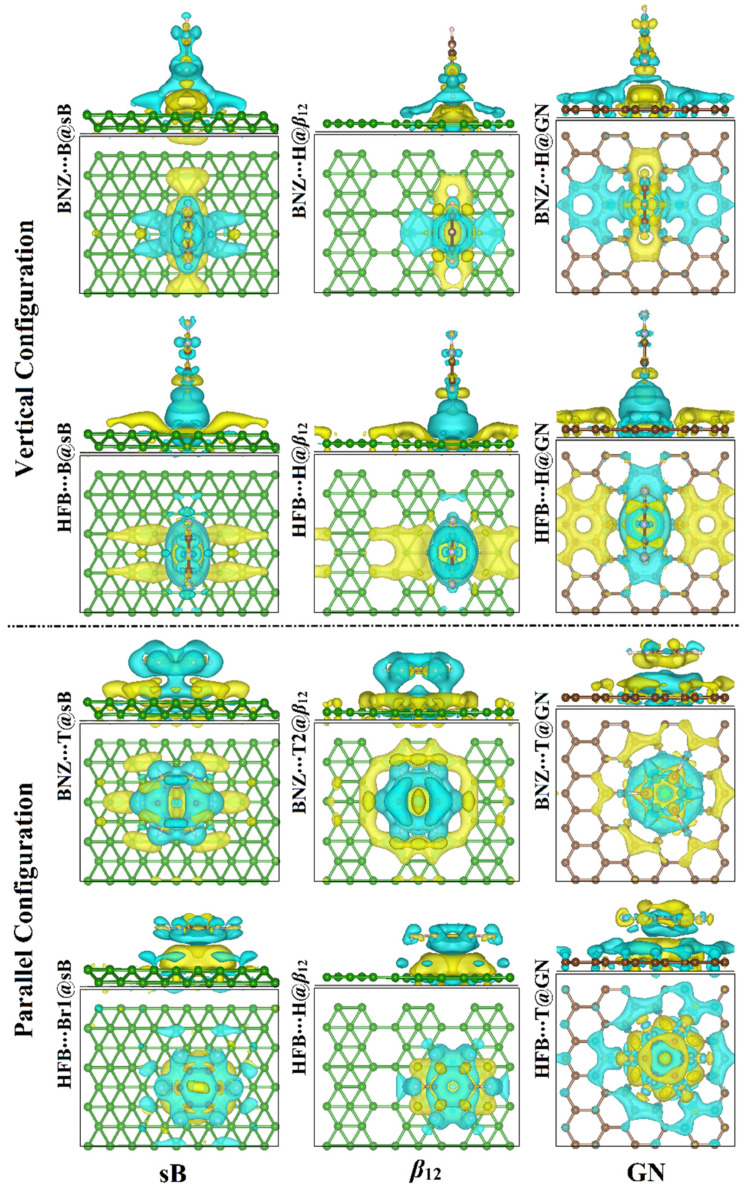
Charge density difference maps of the relaxed structures of the vertical and parallel configurations of the π-system∙∙∙2D sheet complexes where π-system = BNZ/HFB and the 2D sheet = sB/*β*_12_/GN at the most energetically favorable adsorption sites. Cyan and yellow colors represent the charge accumulation (negative) and depletion (positive), respectively. Green, pink, Gray, and brown balls refer to boron, hydrogen, fluorine, and carbon atoms.

To deeply understand the adsorption process, the charge density difference maps for the π-system∙∙∙2D sheet complexes at the most energetically favorable sites were generated based on Equation (3) in the Computational Methods section (Figure 4). With this in mind, the cyan- and yellow-colored-areas referred to increases and decreases in the charge density around the adsorption systems, respectively.

For the π-system∙∙∙2D sheet complexes in the vertical configuration, charge accumulation regions were observed around the BNZ and HFB systems and at the interface sites located over the 2D sheets (Figure 4). According to the latter observation, the charge transfer occurred from the investigated π-systems to the surfaces of the sB, *β*_12_, and GN sheets, confirmed by the negative sign of the estimated *Q*_t_ values (Table 1). Evident accumulated charge regions were denoted by more prominent cyan colors in the case of the adsorption of HFB on the investigated 2D sheets compared with BNZ analog. Accordingly, the 2D sheets exhibited greater potential to attract electrons from HFB than from BNZ analog.

For the BNZ∙∙∙2D sheet complexes in the parallel configuration, the accumulated charge regions were noticed over the 2D sheets, designating the charge transfer from 2D sheets to BNZ. The latter findings were in agreement with the positive *Q*_t_ values listed in Table 1. By contrast, in the case of the adsorption of HFB on the 2D sheets, the electron depletion regions were observed over the 2D sheets, signifying the transfer of charge from HFB to the 2D sheets.

In sum, the current Bader charge analysis generally demonstrated that the π-system∙∙∙sB complexes had the largest charge transfer difference (*Q*_t_) values, followed by the π-system∙∙∙*β*_12_ and, finally, the π-system∙∙∙GN complexes.

### 3.4. Band Structure Calculations

**Figure 5:**
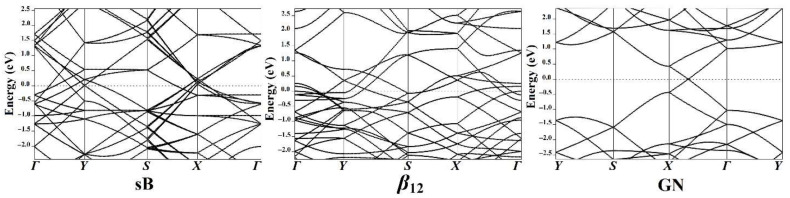
Band structure plots for the pure surfaces of sB, *β*_12_, and GN along the high-symmetry points of the Brillouin zone. The Fermi energy is set to zero-energy.

**Figure 6:**
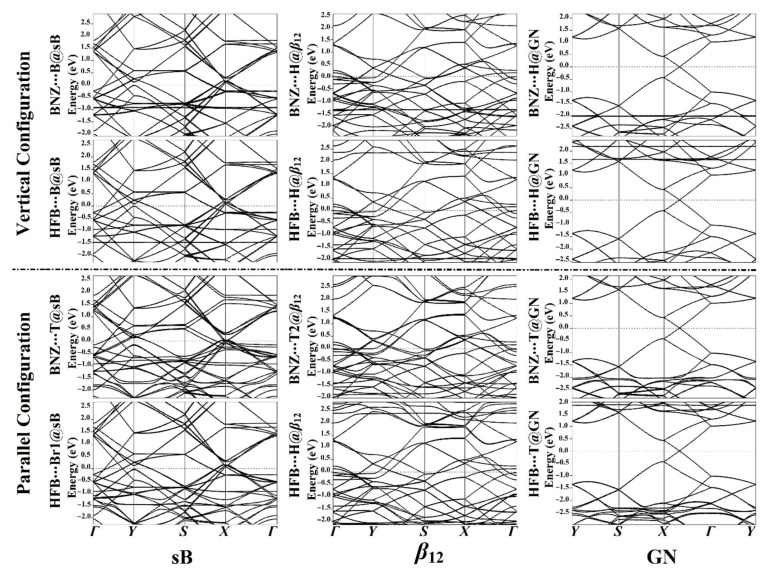
Band structure plots for the sB, *β*_12_, and GN after the adsorption process of BNZ and HFB at the most energetically favorable adsorption sites in the vertical and parallel configurations. The Fermi energy was set to zero-energy.

Electronic band structure analyses were performed to illuminate further the effects of the adsorbed π-systems (i.e., BNZ and HFB) on the electronic properties of the investigated 2D sheets. Using the PBE function, the band structure calculations of the pure 2D sheets were carried out along the high-symmetry points of the Brillouin zone and are depicted in Figure 5. Moreover, the band structures were investigated for the π-system∙∙∙2D sheet complexes at the most energetically favorable adsorption sites to determine the effect of the adsorption process on the electronic properties of the 2D sheets (Figure 6).

As shown in Figure 5, many bands crossing the Fermi level along the *Г-Y-S-X-Г* path were apparently detected in the case of the pure sB and *β*_12_ sheets. Accordingly, the electrons in the valence band could easily move to the conduction band through these bands. In the case of GN surface, the bands along the *Y-S-X-Г-Y* direction met at a point along the Fermi level (i.e., Dirac point), where the bandgap value was zero (Figure 5). Consequently, the findings emphasized that the sB and the *β*_12_ surfaces showed metallic properties, while the GN had semiconductor characters.

For the pure surface of the sB sheet, the bands touched at the *Y*, *Г,* and *X* points at 0.0, −2.0, and 1.6 eV, respectively (Figure 5). Following the adsorption of the BNZ and HFB on the surface of the sB sheet, it was observed that the bands shifted far away from each other, confirming the prominent effect of the adsorption of the π-systems on the electronic properties of the sB sheet (Figure 6). Simultaneously, the adsorption of the BNZ on the sB sheet resulted in the appearance of new valence and conduction bands at roughly −1.0/–2.0 eV, and 1.4/1.7 eV, respectively, demonstrating the interference of the BNZ bands with the sB bands. Extra bands were also found in the valence region of −1.5 and −2.0 eV at *Г* point after the adsorption of HFB on the sB surface in the vertical and parallel configurations.

Similarly, the obtained band structures of the π-system∙∙∙*β*_12_ complexes showed additional bands after the adsorption of the BNZ in both configurations at around −1.5 and −2.0 eV, which might be attributed to the contribution of the orbitals of BNZ in the adsorption process. In comparison, new conduction and valence bands appeared after the adsorption of the HFB in both configurations at 2.3 and −2.0 eV at *Г* point, respectively. Conspicuously, the adsorption of both BNZ and HFB had more valence and conduction bands in the parallel configuration than the vertical configuration, revealing the favorability of the parallel configuration over the vertical configuration. The latter results were in line with the adsorption energy results (Table 1).

The band structure analyses indicated that the adsorption of the electron-rich and electron-deficient π-systems on the GN barely affected the electronic properties of the pure surface and that the Dirac point remained. As shown in Figure 6, the adsorption of BNZ on the surface of the GN in both configurations gave rise to the appearance of a new valence band at −2.0 eV. At the same time, new conduction bands appeared at 1.6 and 2.1 eV for the HFB∙∙∙H@GN complex in the vertical configuration, exhibiting the HFB and GN surface interaction. Overall, the obtained band structures highlighted the occurrence of interactions between the aromatic π-systems and the GN.

### 3.5. Density of State Calculations

**Figure 7:**
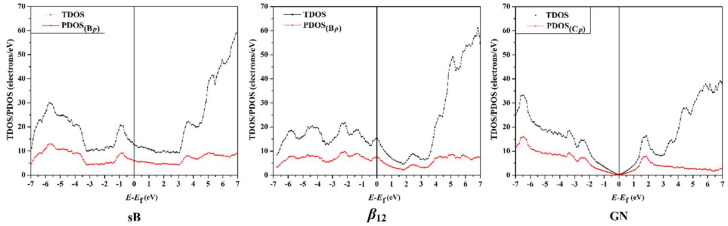
The total and projected density-of-states (TDOS/PDOS) plots for the pure surfaces of the sB, *β*_12_, and GN, assuming the Fermi level as the reference level. The values B*_p_* and C*_p_* represent the *p*-orbital of boron (B) and carbon (C) atoms, respectively.

**Figure 8:**
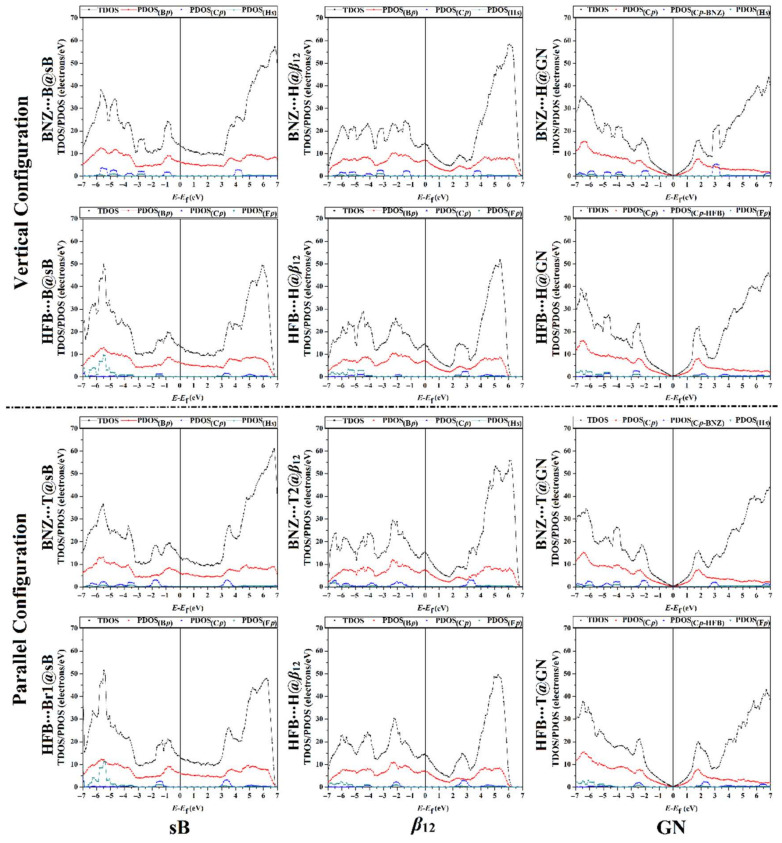
The total and projected density-of-states (TDOS/PDOS) plots for the sB, *β*_12_, and GN after the adsorption process of BNZ and HFB at the most energetically favorable adsorption sites in the vertical and parallel configurations. The B*_p_*/C*_p_*/F*_p_* and H*_s_* represent the *p*-orbital and *s*-orbital of boron (B)/carbon (C)/fluorine (F) and hydrogen (H) atoms, respectively.

The total and projected density-of-states (TDOS/PDOS) analyses were performed to reveal the nature of the interaction between the electron-rich/deficient π-systems and the sB/*β*_12_/GN 2D sheets. The TDOS and PDOS plots were generated for the studied 2D sheets before and after the adsorption process (Figure 7 and Figure 8, respectively).

As illustrated in Figure 7, the TDOS peaks at the Fermi level in the case of both pure sB and *β*_12_ surfaces were not zero, highlighting the metallic property of the examined sheets. In addition, the peaks of the pure sB and *β*_12_ surfaces were found to have a high value of TDOS near the Fermi level, ensuring the conductivity of the surfaces.

With regard to the TDOS of the pure GN surface, there was a Dirac point at the Fermi level (Figure 7), which was consistent with that of the band structure affirmations. From the PDOS results illustrated in Figure 7, the *p*-orbital of the B and C atoms showed the main contribution in the TDOS peaks of the pure sB/*β*_12_ and GN sheets, respectively.

Based on the PDOS peaks of the sB and *β*_12_ surfaces shown in Figure 8, the peaks of the *p*-orbital of the B atoms exhibited the dominant contribution to the TDOS near the Fermi level. In comparison, the *s*-orbital of hydrogen atoms made a frail contribution in the adsorption process.

Notably, the H*_s_*in the BNZ exhibited a minor contribution in the adsorption process on the surface of the sB sheet, with energy values ranging from −6.0 to −2.5 eV in the vertical configuration and from −6.5 to −3.3 eV in the parallel one. Accordingly, the PDOS findings outlined that the adsorption of the BNZ on the sB surface in the vertical and parallel configurations was dominated by the contribution of the C*_p_* in the BNZ. Turning to the HFB∙∙∙sB complexes in both vertical and parallel configurations, the F*_p_*showed the most prevalent contribution to the occurrence of the adsorption process and was found with energy values ranging from −7.0 to −4.5 eV. Remarkably, for the HFB∙∙∙Br1@sB in the parallel configuration, the adsorption process was dominated by the F*_p_* and B*_p_* hybridization, which was located at −5.4 eV. Obviously, the adsorption of HFB on the surface of the sB sheet in the parallel configuration showed higher PDOS peaks than in the vertical configuration, which was in agreement with the adsorption energy results (Table 1).

Concerning the *β*_12_ results, the PDOS analyses of the BNZ∙∙∙H@*β*_12_ and BNZ∙∙∙T2@*β*_12_ complexes in the vertical and parallel configurations, respectively, showed that the C*_p_* in BNZ had the main role in the adsorption process on the *β*_12_ surface. Besides, the H*_s_* made a tiny contribution to the adsorption process, with the PDOS peaks appearing at the valence region below the energy value of −3.0 eV. In addition, the C*_p_* of the HFB displayed considerable hybridization with the B*_p_* in the *β*_12_ sheet, producing a peak in the conduction area at about 3.0 eV. The DOS analyses were compatible with the findings of the adsorption energy and charge transfer calculations (Table 1).

For the GN surface, the adsorption of BNZ at the H@GN and T@GN sites in the vertical and parallel configurations, respectively, originated from the contribution of the C*_p_*of the BNZ, which showed prominent peaks at energy values of −2.0 and 3.0 eV. Prominent C*_p_*and F*_p_* peaks were noted for the adsorption of HFB on the GN surface in both studied configurations, at energy values of −2.5 and 3.0 eV, in the valence and conduction regions, respectively.

Conclusively, the DOS peaks of all the examined π-system∙∙∙2D sheet complexes shifted towards the Fermi level compared to the pure surfaces, highlighting the enhancement of the electrical properties of the investigated 2D sheets after the adsorption process.

## 4. Conclusions

In the current study, the adsorption of electron-rich and electron-deficient π-systems (BNZ and HFB) on borophene and graphene sheets was comparatively investigated using DFT calculations. Adsorption energies, charge transfer analyses, electronic band structures, and DOS calculations were performed for the π-system∙∙∙2D sheet in the vertical and parallel configurations. The BNZ and HFB adsorption on the sB, *β*12, and GN surfaces in the parallel configuration demonstrated more favorable amplitude than that in the vertical configuration. The adsorption of BNZ and HFB over the sB and *β*12 sheets enhanced the electronic properties of their surfaces. On the other hand, the adsorption of BNZ and HFB on the GN sheet exhibited nominal change in the electronic properties of the GN surface. Based on Bader charge analysis, the charge was transferred from BNZ and HFB to the investigated 2D sheets, with the exception of the adsorption of BNZ in the parallel configuration, in which the charge was transferred from the 2D sheets to BNZ. Crucially, the band structure and the density-of-states (DOSs) results confirmed that the adsorption process of the π-system on the surface of the investigated 2D sheets bolstered their electrical properties. The results of this research could provide a base of information for fields relevant to the application of 2D nanomaterials.

## Figures and Tables

**Table 1:** Adsorption energies (*E*_ad_, kcal/mol) and equilibrium distances (d, Å) of the relaxed structures of BNZ and HFB on the sB, *β*_12_, and GN sheets in the vertical and parallel configurations at all studied adsorption sites, as well as the charge transfer difference (*Q*_t_, e) for the 2D sheets before and after the adsorption process.

2D Sheet	Adsorption Site ^a^	π-System					
		Benzene (BNZ)			Hexafluorobenzene (HFB)		
		*E*_ad_ (kcal/mol)	d (Å)	*Q*_t_^b^ (e)	*E*_ad_ (kcal/mol)	d (Å)	*Q*_t_^b^ (e)
**Vertical Configuration ^c^**							
sB	T	−4.35	2.57	−0.0154	−3.99	2.95	−0.0275
	B	−5.66	3.02	−0.0136	−4.83	3.52	−0.0318
	Br1	−5.64	3.02	−0.0132	−4.79	3.53	−0.0318
	Br2	−4.40	2.54	−0.0169	−4.00	2.93	−0.0274
*β*12	T1	−5.07	2.54	−0.0176	−3.94	3.02	−0.0219
	T2	−4.35	2.62	−0.0157	−4.39	2.94	−0.0219
	T3	−4.84	2.53	−0.0165	−4.22	2.96	−0.0220
	H	−6.40	2.15	−0.0199	−4.54	2.72	−0.0274
	Br1	−5.65	2.38	−0.0177	−4.20	2.90	−0.0261
	Br2	−4.95	2.47	−0.0157	−4.50	2.91	−0.0215
GN	T	−4.30	2.52	−0.0067	−3.74	2.89	−0.0124
	H	−5.04	2.27	−0.0058	−3.97	2.79	−0.0117
	Br	−4.47	2.45	−0.0074	−3.76	2.88	−0.0121
**Parallel Configuration ^c^**							
sB	T	−14.93	3.09	0.0735	−14.66	3.23	−0.0733
	B	−11.46	4.00	0.0469	−15.74	4.04	−0.0812
	Br1	−11.43	4.03	0.0454	−15.76	4.06	−0.0813
	Br2	−14.50	3.01	0.0572	−14.75	3.18	−0.0730
*β*_12_	T1	−11.45	3.26	0.0184	−16.18	3.27	−0.0672
	T2	−12.16	3.24	0.0436	−16.05	3.27	−0.0520
	T3	−10.50	3.30	0.0066	−15.80	3.27	−0.0652
	H	−11.73	3.23	0.0385	−16.55	3.23	−0.0534
	Br1	−11.55	3.25	0.0184	−16.19	3.22	−0.0608
	Br2	−10.77	3.30	0.0099	−15.66	3.27	−0.0645
GN	T	−9.97	3.28	0.0065	−14.34	3.21	−0.0378
	H	−8.78	3.40	0.0080	−12.51	3.33	−0.0325
	Br	−9.89	3.28	0.0050	−14.08	3.22	−0.0357

^a^ All adsorption sites on the surface of the studied 2D sheets are illustrated in Figure 2. ^b^$\;Q_{t}$ was estimated by Equation (2) (see Computational Methods section for details). ^c^ All the relaxed structures of the π-system∙∙∙2D sheet complexes are displayed in Figure S1.

## Data Availability

Not applicable.

## Supplementary Materials

The following supporting information can be downloaded at: https://www.mdpi.com/article/10.3390/nano12061028/s1. Figure S1: Side and top views of the relaxed structures of the vertical and parallel configurations of the π-system∙∙∙2D sheet complexes where π-system = benzene (BNZ)/hexafluorobenzene (HFB) and the 2D sheet = striped borophene (sB)/*β*_12_ borophene (*β*_12_)/pristine graphene (GN) at all possible adsorption sites.
