# Supplementary material for: Borophene and Pristine Graphene 2D Sheets as Potential Surfaces for the Adsorption of Electron-Rich and Electron-Deficient π-Systems: A Comparative DFT Study
Source: Nanomaterials (Basel). 2022 Mar 21;12(6):1028. doi: 10.3390/nano12061028 (PMC8948930; doi:10.3390/nano12061028)
Supplement: Supplementary file 1 [file nanomaterials-12-01028-s001.zip › nanomaterials-1635101-supplementary.pdf]

nanomaterials

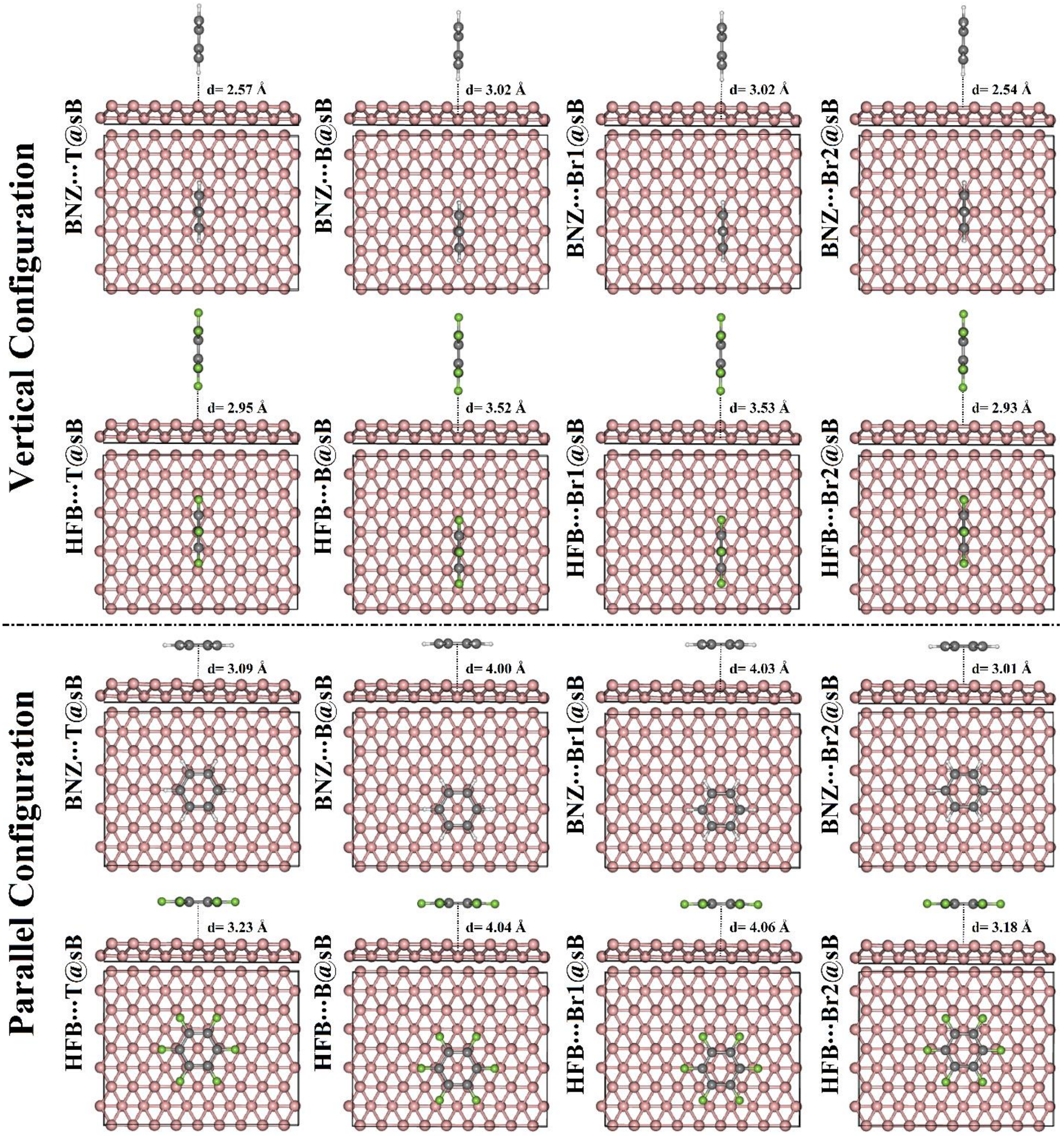

Figure S1. Side and top views of the relaxed structures of the vertical and parallel configurations of the π-system···2D sheet complexes where π-system = benzene (BNZ)/hexafluorobenzene (HFB) and the 2D sheet = striped borophene (sB)/β_{12} borophene (β_{12})/pristine graphene (GN) at all possible adsorption sites. The π-system···2D sheet equilibrium distances (d) are given in Å.

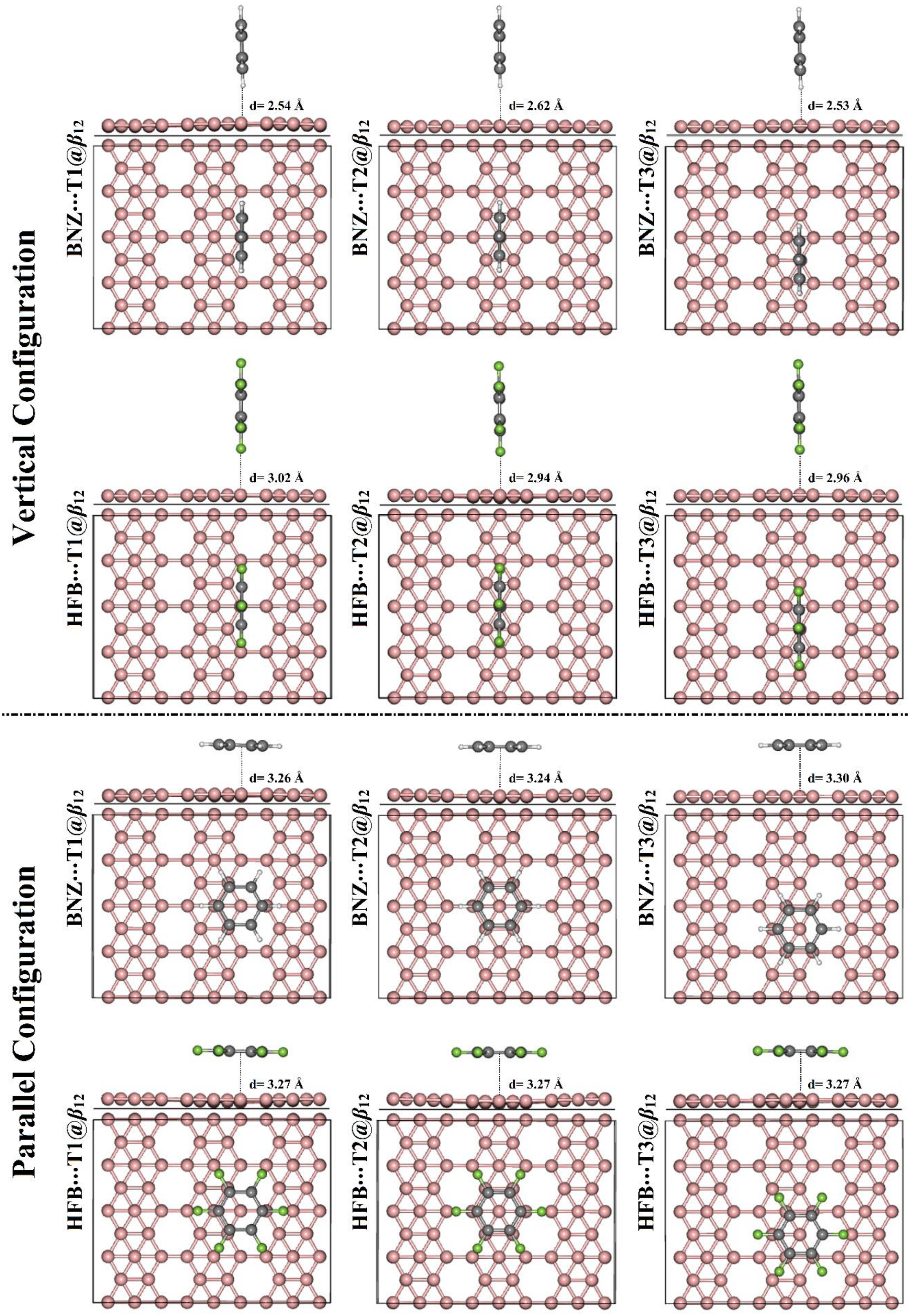

Figure S1. Continued.

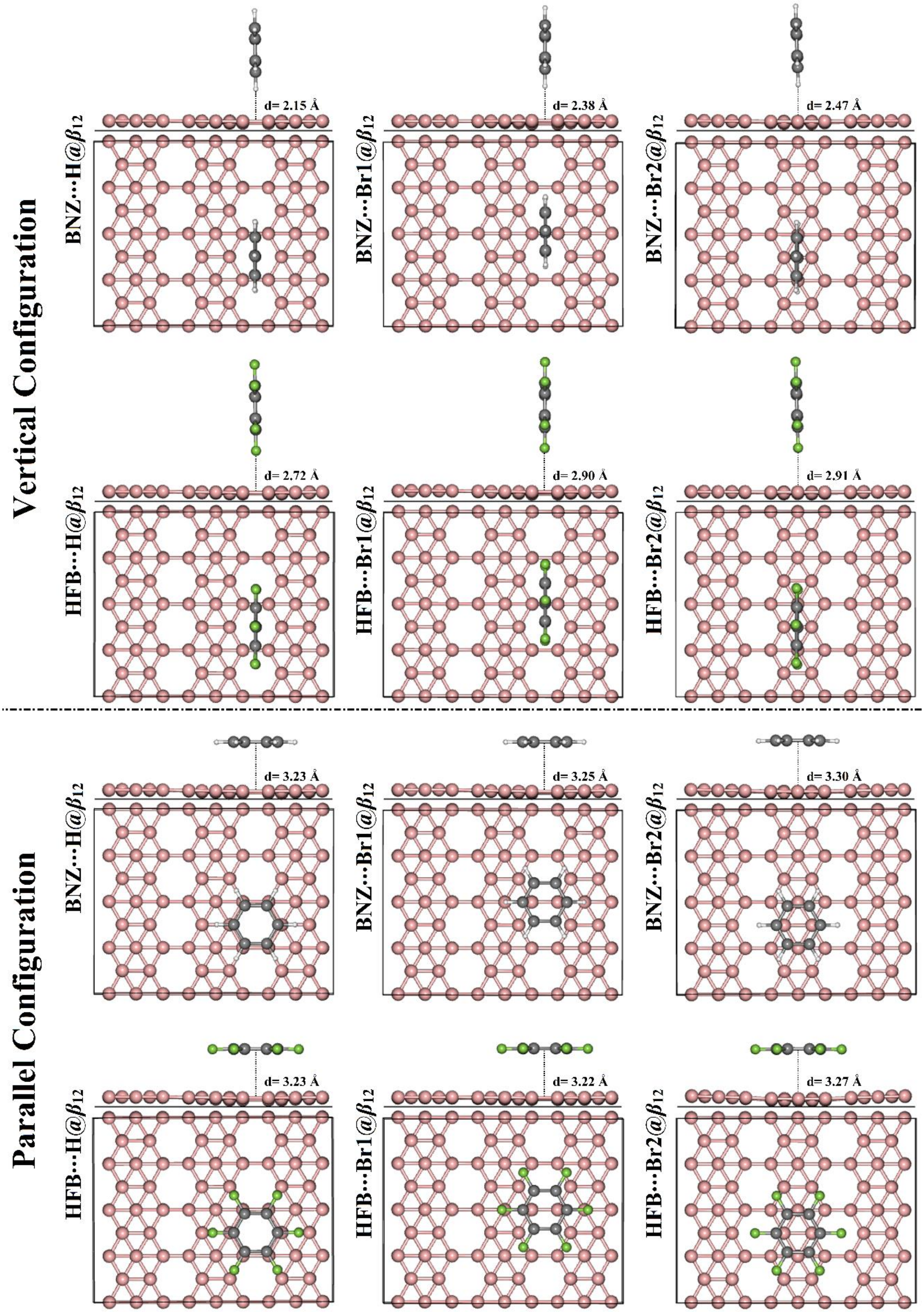

Figure S1. Continued.

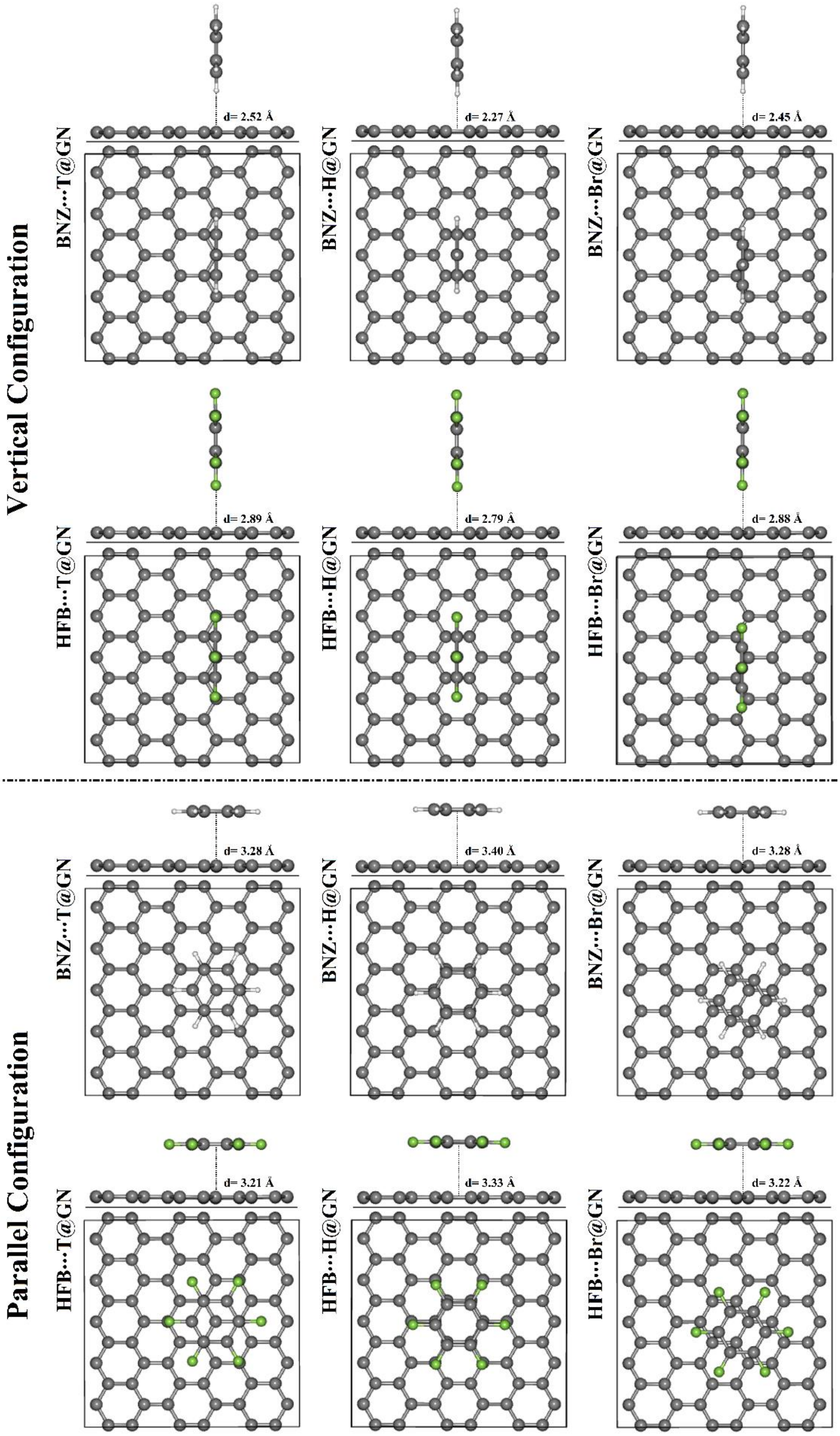

Figure S1. Continued.
